# 2415. Health Outcome Disparities in Device-Associated Healthcare Infections Before and During the COVID-19 Pandemic

**DOI:** 10.1093/ofid/ofad500.2035

**Published:** 2023-11-27

**Authors:** Werner Bischoff, Joni Evans, Catherine Passaretti, Andrea Fernandez

**Affiliations:** Wake Forest University School of Medicine, Winston Salem, NC; Wake Forest University School of Medicine, Winston Salem, North Carolina; Advocate Health, Charlotte, NC; Atrium Health Wake Forest Baptist, Winston Salem, North Carolina

## Abstract

**Background:**

The COVID-19 pandemic has led to a considerable increase in Central-Line Associated Bloodstream Infections (CLABSI) and Catheter Associated Urinary Tract Infections (CAUTI). However, it remains unclear how this affected different population groups.

**Methods:**

This retrospective observational cohort study included CLABSI and CAUTI in a tertiary care facility. Information was collected on patient demographics, hospitalization, comorbidities, and COVID-19 status. Chi-square and Wilcoxon rank-sum tests were used for categorical and continuous variable comparisons. GEE models compared pre- and pandemic periods by interrupted time series analysis.

**Results:**

From 1/1/2018 to 5/31/2022 98,791 patients had 151,550 hospital admissions. Of those, 17,796 patients had 29,483 central lines placed and 45,180 patients had 65,422 Foleys. 314 patients developed 338 CLABSI and 216 patients had 217 CAUTI. 1,552 patients tested positive for COVID-19 with 22 developing CLABSI and 14 CAUTI. The pre-pandemic downward trend in CLABSI and CAUTI was reversed during COVID-19 (p< 0.05).

Black patients and those with other/unknown race had higher CLABSI per patient with central lines (p< 0.02) and higher device days (p< 0.0001) compared to white patients. Hispanic/Latino patients acquired more CLABSI per patient compared to non-Hispanic/non-Latino patients (p=0.04). During COVID-19 the already higher device days in Hispanic/Latino patients further increased compared to non-Hispanic/non-Latino patients (p< 0.0001).

Black patients had higher CAUTI infection rates per patient with Foleys compared to white and other/unknown patients (p< 0.05). CAUTI per device days were also higher in black patients compared to white patients (p=0.02). No difference in CAUTI burden was detected for ethnic groups throughout the study period. Black and non-Hispanic/non-Latino patients had higher Foley usage days than other patients (p< 0.0001). Foley days decreased during COVID-19 (p=0.01).

**Conclusion:**

We detected health outcome disparities affecting black (CLABSI and CAUTI) and Hispanic/Latino (CLABSI) patients. Infections increased during the pandemic without altering race/ethnicity differences. Device utilization was significantly higher in affected groups.

**Disclosures:**

**All Authors**: No reported disclosures

